# Keratometric Index Obtained by Fourier-Domain Optical Coherence Tomography

**DOI:** 10.1371/journal.pone.0122441

**Published:** 2015-04-17

**Authors:** Yanjun Hua, Aleksander Stojanovic, Tor Paaske Utheim, Xiangjun Chen, Sten Ræder, Jinhai Huang, Qinmei Wang

**Affiliations:** 1 Department of Ophthalmology, Shanghai Jiao Tong University Affiliated Sixth People's Hospital, Shanghai, 200233, China; 2 Department of Ophthalmology, University Hospital of North Norway, Tromsø, Norway; 3 SynsLaser Kirurgi AS, Tromsø/Oslo, Norway; 4 Department of Medical Biochemistry, Oslo University Hospital, Oslo, Norway; 5 School of Optometry and Ophthalmology and Eye Hospital, Wenzhou Medical University, Key Laboratory of Vision Science, Ministry of Health of People’s Republic of China, Wenzhou, Zhejiang, People’s Republic of China; Save Sight Institute, AUSTRALIA

## Abstract

**Purpose:**

To determine the keratometric indices calculated based on parameters obtained by Fourier-domain optical coherence tomography (FD-OCT).

**Methods:**

The ratio of anterior corneal curvature to posterior corneal curvature (Ratio) and keratometric index (N) were calculated within central 3 mm zone with the RTVue FD-OCT (RTVue, Optovue, Inc.) in 186 untreated eyes, 60 post-LASIK/PRK eyes, and 39 keratoconus eyes. The total corneal powers were calculated using different keratometric indices: K_cal_ based on the mean calculated keratometric index, K_1.3315_ calculated by the keratometric index of 1.3315, and K_1.3375_ calculated by the keratometric index of 1.3375. In addition, the total corneal powers based on Gaussian optics formula (K_actual_) were calculated.

**Results:**

The means for Ratio in untreated controls, post-LASIK/PRK group and keratoconus group were 1.176 ± 0.022 (95% confidence interval (CI), 1.172–1.179), 1.314 ± 0.042 (95%CI, 1.303–1.325) and 1.229 ± 0.118 (95%CI, 1.191–1.267), respectively. And the mean calculated keratometric index in untreated controls, post-LASIK/PRK group and keratoconus group were 1.3299 ± 0.00085 (95%CI, 1.3272–1.3308), 1.3242 ± 0.00171 (95%CI, 1.3238–1.3246) and 1.3277 ± 0.0046 (95%CI, 1.3263–1.3292), respectively. All the parameters were normally distributed. The differences between K_cal_ and K_actual_, K_1.3315_ and K_actual_, and K_1.3375_ and K_actual_ were 0.00 ± 0.11 D, 0.21 ± 0.11 D and 0.99 ± 0.12 D, respectively, in untreated controls; -0.01 ± 0.20 D, 0.85 ± 0.18 D and 1.56 ± 0.16 D, respectively, in post-LASIK/PRK group; and 0.03 ± 0.67 D, 0.56 ± 0.70 D and 1.40 ± 0.76 D, respectively, in keratoconus group.

**Conclusion:**

The calculated keratometric index is negatively related to the ratio of anterior corneal curvature to posterior corneal curvature in untreated, post-LASIK/PRK, and keratoconus eyes, respectively. Using the calculated keratometric index may improve the prediction accuracies of total corneal powers in untreated controls, but not in post-LASIK/PRK and keratoconus eyes.

## Introduction

Consideration of the keratometric index is essential for the assessment of total corneal power and the prediction of intraocular lens power.[1,2] Historically, because of the lack of information from the posterior corneal surface, the conventional keratometric index (1.3375)[3] or the keratometric index derived from Gullstrand schematic eye (1.3315)[4] combining the anterior corneal curvature were used to calculate the total corneal power. The equation is as follows: K = (N-1) / R, where K is the total corneal power, N is the keratometric index, 1 means the refractive index of the air, and R means the anterior corneal curvature in certain central zone. In theory, these keratometric indices (the conventional 1.3375 or the 1.3315 based on the Gullstrand schematic eye) which are lower than the real corneal refractive index of 1.376, compensate for the negative power of posterior corneal surface. Moreover, the equation above must be based on 2 assumptions: The anterior and posterior corneal curvatures have a constant and linear relationship.[5,6] However, this is not the case. Even in different schematic eyes, the ratio of anterior corneal curvature to posterior corneal curvature are not the same.[6] Furthermore, the ratios obtained by different devices from real human eyes are also vary.[7–9]

Currently, there are three kinds of techniques that can detect the information of the anterior and posterior corneal surface and central corneal thickness (CCT) simultaneously, including Slit-scan system (Orbscan Ⅱ) [10,11], Scheimpflug camera system (Pentacam or Pentacam HR)[12,13] and optical coherence tomography system (Fourier-Domain OCT, FD-OCT)[14–16]. There have been reports in which the first 2 techniques were applied to obtain the ratio of anterior corneal curvature to posterior corneal curvature, and the keratometric index was calculated regarding the total corneal power based on Gaussian optics formula as a benchmark.[1,5,6,17] However, to the best to our knowledge, until now there have been no reports on the keratometric index calculated by data derived from RTVue FD-OCT (RTVue, Optovue, Inc.).

The commercially available system RTVue FD-OCT with a speed of 26000 axial scans per second has an axial resolution of 5 μm and a transverse resolution of 15 μm.[16] The corneal mapping model had 6.0 mm line scans on 8 meridians with 1019 axial scans centered on the pupil, and the whole scan model was finished within 0.32 seconds.[16] Previous work from our research group showed that RTVue FD-OCT had good repeatability in measurements of corneal parameters (corneal curvature and CCT) from untreated eyes and eyes after corneal refractive surgery.[18,19] In this study, we used the RTVue FD-OCT system to measure the anterior and posterior corneal curvature and the CCT within central 3 mm zone in untreated controls, post-LASIK/PRK and keratoconus groups. The ratio of anterior corneal curvature to posterior corneal curvature and keratometric indices were then calculated, and the total corneal powers were assessed using these keratometric indices in each group.

## Patients and Methods

Inclusion criteria were patients who didn’t receive prior corneal or ocular surgery in the untreated controls, or received uneventful LASIK at least 3 months or PRK at least 6 months previously in the post-LASIK/PRK group, or was diagnosed with keratoconus in the keratoconus group. All the results obtained by RTVue FD-OCT had good reliability (Measurement Reliability Rating GOOD displayed on the Pachymetry + CPwr map). Three groups of subjects were included in this study:

1. 186 eyes of 186 subjects in the untreated group; the mean age was 26.1 ± 5.6 years; the mean spherical equivalent was -2.53 ± 1.32 D; these subjects were selected from the patients examined before corneal refractive surgery in Wenzhou Eye Hospital (Wenzhou, China) from July 2011 to April 2012.
2. 60 eyes of 39 patients in the post-LASIK/PRK group; the mean age was 27.3 ± 6.2 years; the mean spherical equivalent before the laser surgery was -4.81±1.22 D; the mean spherical equivalent after the laser surgery was 0.02±0.33 D; these subjects were selected from the patients who received LASIK at least 3 months previously or PRK at least 6 month previously in Wenzhou Eye Hospital from July 2011 to April 2012.
3. 39 eyes of 27 patients in the keratoconus group; the mean age was 34.93 ± 11.41 years; the data were selected from the patients who were diagnosed as keratoconus in Synslaser Clinic(Tromso, Norway) from September 2012 to January 2013. All the patients did not receive any eye surgery previously. And 4 of them had apical scars, 5 of them had Vogt striae, and all the others had clean corneas.

The study was conducted at the Eye Hospital of Wenzhou Medical University and Synslaser Clinic (Tromso, Norway). The research was performed in accordance with the principles stated in the Declaration of Helsinki. The untreated controls and post-LASIK/PKR group from Wenzhou Eye Hospital was approved by the Office of Research Ethical Committee, Wenzhou Medical University; and the keratoconus group from Synslaser was approved by the National Committee for Medical and Health Research Ethics in Norway. All participants provided written informed consent after the nature of the study had been explained to them.

We obtained the anterior corneal curvature (R_anterior_), posterior corneal curvature (R_posterior_) and CCT within central 3 mm zone for each tested eye using RTVue FD-OCT. The ratio of the anterior and posterior corneal curvature in each eye was calculated as follows:

$$\text{Ratio}={\text{R}}_{\text{anterior}}/{\text{R}}_{\text{posterior}}$$ 1

The Gaussian optics formula calculates the actual total corneal power (K_actual_) within central 3 mm zone by assuming the central cornea as a thick lens. And K_actual_ in each eye was calculated as follows:

$$\begin{array}{l} {\text{K}}_{\text{actual}}=\left({\text{n}}_{1}–{\text{n}}_{0}\right)/{\text{R}}_{\text{anterior}}+\left({\text{n}}_{2}–{\text{n}}_{1}\right)/{\text{R}}_{\text{posterior}}–\left(\text{CCT}/{\text{n}}_{1}\right)\times \left[\left({\text{n}}_{1}–{\text{n}}_{0}\right)/{\text{R}}_{\text{anterior}}\right]\times \\ \left[\left({\text{n}}_{2}–{\text{n}}_{1}\right)/{\text{R}}_{\text{posterior}}\right] \end{array}$$ 2

where n_0_ (1.000), n_1_ (1.376) and n_2_ (1.336) are the refractive indices of the air, the cornea and aqueous, respectively. We used the following equation to compute the real keratometric index (n) in each eye:

$$\left(\text{n}-1\right)/{\text{R}}_{\text{anterior}}={\text{K}}_{\text{actual}}$$ 3

The mean of n in each eye was computed and defined as N, next N was used to estimate the total corneal power (K_cal_) in each eye as follows:

$${\text{K}}_{\text{cal}}=\left(\text{N}-1\right)/{\text{R}}_{\text{anterior}}$$ 4

The total corneal power estimated using the keratometric index obtained from Gullstrand model eye (1.3315) and the conventional keratometric index (1.3375) were calculated as follows:

$${\text{K}}_{1.3315}=\left(1.3315-1\right)/{\text{R}}_{\text{anterior}}$$ 5

$${\text{K}}_{1.3375}=\left(1.3375-1\right)/{\text{R}}_{\text{anterior}}$$ 6

All the data were entered into MedCalc Version 11.4.2 for Windows. Correlation coefficient and paired t-test were used to compare values normally distributed in the three groups. Correlation coefficient and regression analysis were used to analyze the correlations between R_anterior_ and R_posterior_, N_cal_ and Ratio. Bland-Altman plots were applied to analyze the agreement between K_cal_ and K_actual_, K_1.3315_ and K_actual_, K_1.3375_ and K_actual_. A *P* value of 0.05 or less was considered statistically significant.

## Results

**Fig 1:**
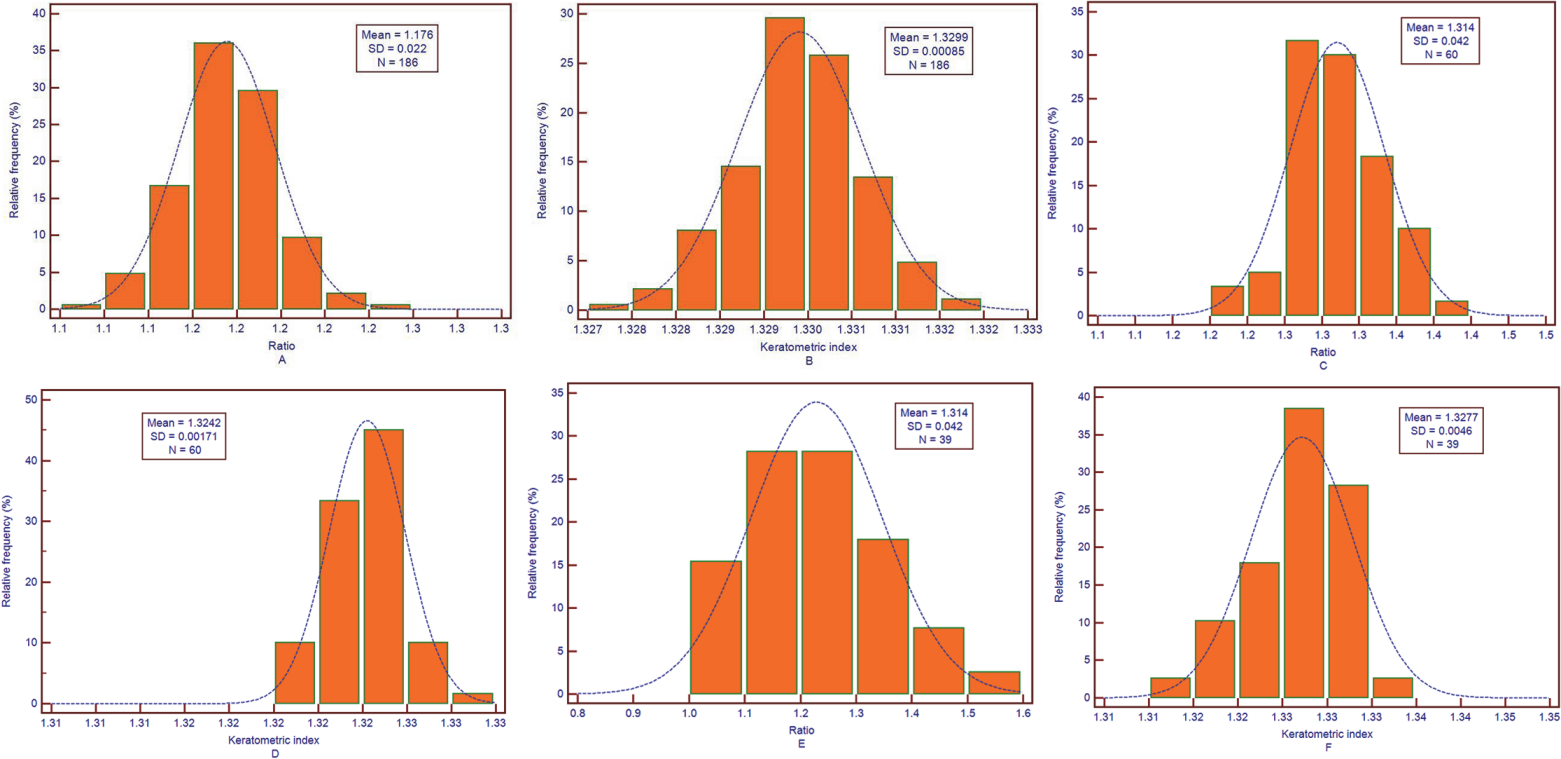
The distribution of Ratio and keratometric index (N) in the three group. A: The distribution of Ratio (the ratio of the anterior corneal curvature to the posterior corneal curvature in central 3 mm zone) conforms to a normal distribution (*P* = 0.946, Kolmogorov-Smirnov test) in untreated controls. B: The distribution of calculated keratometric index (N) conforms to a normal distribution (*P* = 0.992, Kolmogorov-Smirnov test) in untreated controls. C: The distribution of Ratio conforms to a normal distribution (*P* = 0.945, Kolmogorov-Smirnov test) in post-LASIK/PRK group. D: The distribution of calculated keratometric index (N) conforms to a normal distribution (*P* = 0.972, Kolmogorov-Smirnov test) in post-LASIK/PRK group. E: The distribution of Ratio conforms to a normal distribution (*P* = 0.884, Kolmogorov-Smirnov test) in keratoconus group. F: The distribution of calculated keratometric index (N) conforms to a normal distribution (*P* = 0.888, Kolmogorov-Smirnov test) in keratoconus group.

Table 1 shows the corneal parameters obtained by RTVue FD-OCT in untreated controls, post-LASIK/PRK group and keratoconus group. All the values conformed to be normally distributed by Kolmogorov-Smirnov test (all *P* > 0.05). The distributions of Ratio and keratometric indices (N) in untreated controls, post-LASIK/PRK group and keratoconus group are shown in Fig 1A, 1B, 1C, 1D, 1E and 1F, respectively.

**Table 1:** Summary of corneal parameters in untreated controls, post-LASIK/PRK and keratoconus group.

Parameter	Mean(D)±SD	95% CI	Range	*P* Value*
Untreated controls				
R_anterior_ (mm)	7.718 ± 0.275	7.678–7.758	7.016–8.561	0.994
R_posterior_ (mm)	6.569 ± 0.265	6.531–6.608	5.849–7.259	0.674
CCT (μm)	544.98 ± 33.09	540.82–550.10	433.33–629.33	0.442
Ratio	1.176 ± 0.022	1.172–1.179	1.12–1.25	0.946
N	1.3299 ± 0.00085	1.3272–1.3308	1.3271–1.3321	0.992
Post-LASIK/PRK group				
R_anterior_ (mm)	8.582 ± 0.311	8.501–8.662	8.050–9.283	0.803
R_posterior_ (mm)	6.534 ± 0.164	6.491–6.576	6.158–6.844	0.695
CCT (μm)	451.09 ± 33.56	442.42–459.76	374.00–536.00	0.425
Ratio	1.314 ± 0.042	1.303–1.325	1.210–1.406	0.945
N	1.3242 ± 0.00171	1.3238–1.3246	1.3204–1.3285	0.972
Keratoconus group				
R_anterior_(mm)	7.214 ± 0.647	7.004–7.424	5.588–8.732	0.500
R_posterior_(mm)	5.948 ± 0.924	5.648–6.247	3.721–7.395	0.692
CCT(μm)	480.23 ± 41.93	466.64–493.82	385–572	0.860
Ratio	1.229 ± 0.118	1.191–1.267	1.050–1.502	0.884
N	1.3277 ± 0.0046	1.3263–1.3292	1.3171–1.3348	0.888

R_anterior_ = the anterior corneal curvature within 3 mm zone; R_posterior_ = the posterior corneal curvature within 3 mm zone; Ratio = R_anterior_ / R_posterior_; CCT = central corneal thickness within 3 mm zone; N = calculated keratometric index based on Gaussian thick lens formula; CI = consistent interval.

*Kolmogorov-Smirnov test.

**Fig 2:**
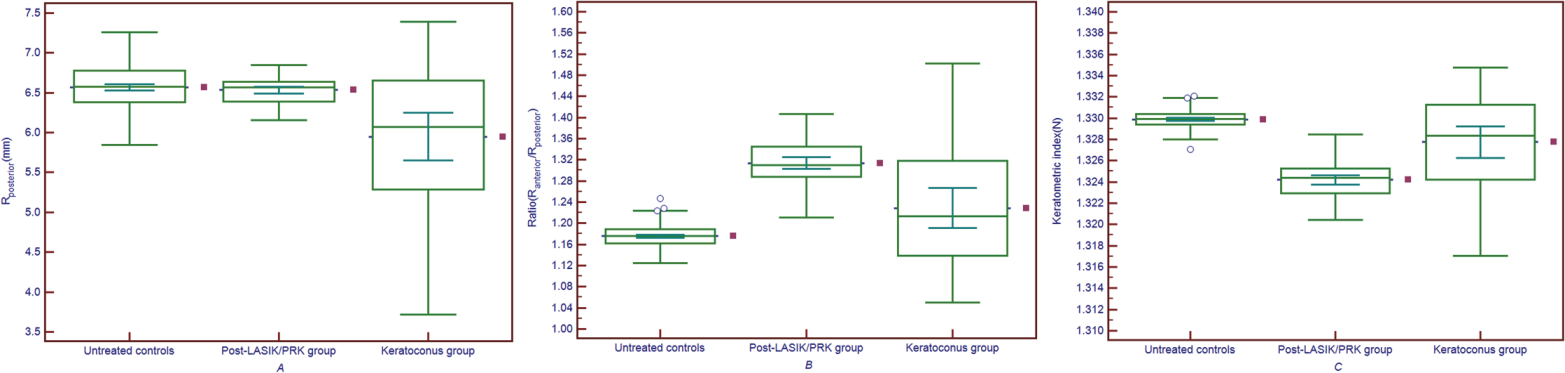
The multiple variables graphs of R_posterior_ values, Ratio and keratometric index (N) from the three group. *A*: The multiple variables graphs of R_posterior_ values from untreated controls, post-LASIK/PRK group and keratoconus group. *B*: the multiple variables graphs of Ratio values from untreated controls, post-LASIK/PRK group and keratoconus group. *C*: The multiple variables graphs of calculated keratometric index (N) from untreated controls, post-LASIK/PRK group and keratoconus group.

Fig 2 shows the multiple variable graphs of the mean R_posterior_, Ratio and Keratometric index (N) in untreated controls, post-LASIK/PRK group and keratoconus group. In Fig 2*A*, the mean R_posterior_ from keratoconus group was significantly smaller than those from untreated and post-LASIK/PRK group (both *P*<0.05). And there was no statistical significance between the mean R_posterior_ values from untreated controls and post-LASIK/PRK group (*P*>0.05). In Fig 2*B*, both the mean Ratio from post-LASIK/PRK group and keratoconus group were larger than the mean Ratio from untreated controls (both *P*<0.05), and the mean Ratio from post-LASIK/PRK group was larger than that from keratoconus group (*P*<0.05). In Fig 2*C*, both the mean keratometric indices (N_cal_) from post-LASIK/PRK group and keratoconus group were smaller than the mean Ratio from untreated controls (both *P*<0.05), and the mean N from post-LASIK/PRK group was smaller than that from keratoconus group (*P*<0.05).

**Fig 3:**
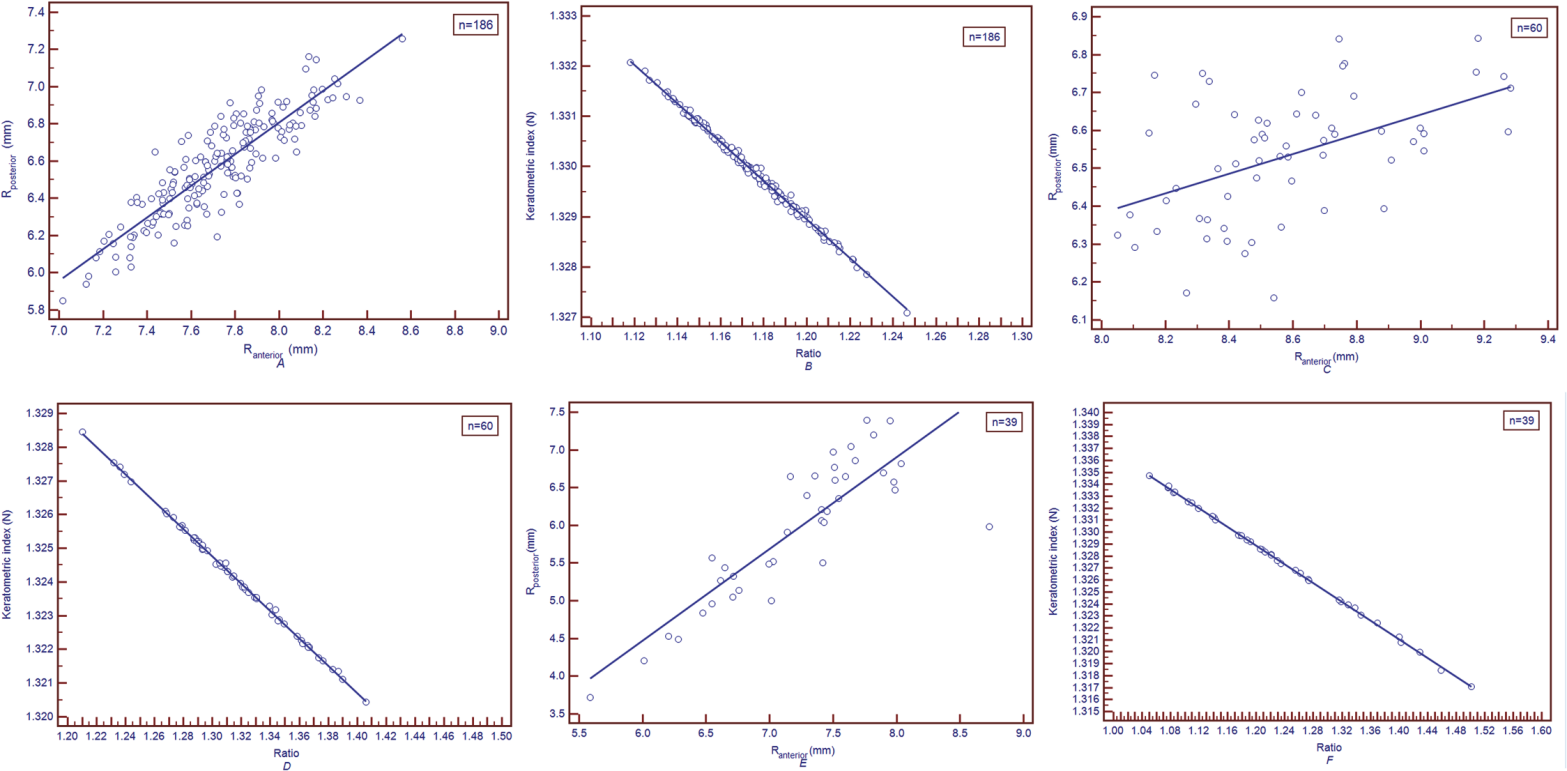
The scatter diagram & regression line of R_anterior_ and R_posterior_, Ratio and keratometric index (N) in the three group. *A*: The scatter diagram & regression line of R_anterior_ and R_posterior_ in untreated controls. The regression equation was R_posterior_ = 0.01021 + 0.8499×R_anterior_ (*r* = 0.884, *r2* = 0.782, *P*<0.05). *B*: The scatter diagram & regression line of Ratio and keratometric index (N) in untreated controls. The regression equation was N = 1.3752–0.03852×Ratio (*r* = -0.997, *r2* = 0.995, *P*<0.05). *C*: The scatter diagram & regression line of R_anterior_ and R_posterior_ in post-LASIK/PRK group. The regression equation was R_posterior_ = 4.3054 + 0.2596×R_anterior_ (*r* = 0.492, *r* ^2^ = 0.242, *P*<0.05). *D*: The scatter diagram & regression line of Ratio and keratometric index (N) in post-LASIK/PRK group. The regression equation was N = 1.3775–0.04054×Ratio (*r* = -0.999, *r2* = 0.999, P<0.05). *E*: The scatter diagram & regression line of R_anterior_ and R_posterior_ in keratoconus group. The regression equation was R_posterior_ = -2.8393 + 1.218×R_anterior_ (*r* = 0.854, *r2* = 0.728, *P*<0.05). *F*: The scatter diagram & regression line of Ratio and keratometric index (N) in keratoconus group. The regression equation was N = 1.3759–0.03917×Ratio (*r* = -0.999, *r2* = 0.999, *P*<0.05).

In untreated controls, linear regression revealed that there were good correlations between R_anterior_ and R_posterior_, Ratio and N. The regression equations were R_posterior_ = 0.01021 + 0.8499×R_anterior_ (*r* = 0.884, *r* ^*2*^ = 0.782, *P*<0.05) and N = 1.3752–0.03852×Ratio (*r* = -0.997, *r* ^*2*^ = 0.995, P<0.05) in Fig 3*A* and 3*B*. In post-LASIK/PRK group, linear regression revealed that there were good correlation between Ratio and N. The regression equations was N = 1.3775–0.04054×Ratio (*r* = -0.999, *r* ^*2*^ = 0.999, P<0.05) in Fig 3*D*. In keratoconus group, the regression equations were R_posterior_ = -2.8393 + 1.218×R_anterior_ (*r* = 0.854, *r* ^*2*^ = 0.728, *P*<0.05) and N = 1.3759–0.03917×Ratio (*r* = -0.999, *r* ^*2*^ = 0.999, P<0.05) in Fig 3*E* and 3*F*.

Table 2 shows the total corneal power calculated with different keratometric indices in untreated controls, post-LASIK/PRK group and keratoconus group. Table 3 shows the comparisons between K_cal_ and K_actual_, K_1.3315_ and K_actual_, K_1.3375_ and K_actual_ in the three groups. There were no statistical significances between K_cal_ and K_actual_ in all three groups (all *P* = 1.00, paired *t* test). In untreated controls, K_1.3315_ and K_1.3375_ were 0.21 ± 0.11 D and 0.99 ± 0.12 D lager than K_actual_, respectively (both *P*<0.05).

**Table 2:** Mean values and 95%CI of total corneal power calculated with different keratometric indices in untreated controls, post-LASIK/PRK and keratoconus group.

	Untreated controls	Post-LASIK/PRK group	Keratoconus group
K_actual_	42.80 ± 1.53 (42.58 to 43.02)	37.83 ± 1.50 (37.44 to 38.22)	45.77 ± 3.93 (44.50 to 47.05)
K_cal_	42.80 ± 1.53 (42.58 to 43.02)	37.83 ± 1.35 (37.48 to 38.17)	45.80 ± 4.33 (44.40 to 47.20)
K_1.3315_	43.01 ± 1.53 (42.78 to 43.23)	38.67 ± 1.38 (38.32 to 39.04)	46.33 ± 4.38 (44.91 to 47.75)
K_1.3375_	43.79 ± 1.56 (43.56 to 44.01)	39.38 ± 1.40 (39.02 to 39.74)	47.17 ± 4.46 (45.73 to 48.61)

K_actual_ = actual total corneal power calculated based on Gaussian optics formula; K_cal_ = total corneal power calculated with the mean keratometric indices (1.3299 in untreated controls, 1.3242 in post-LASIK/PRK group and 1.3277 in keratoconus group); K_1.3315_ = total corneal power calculated with the keratometric index of 1.3315; K_1.3375_ = total corneal power calculated with the keratometric index of 1.3375.

**Table 3:** The differences between K_cal_ and K_actual_, K_1.3315_ and K_actual_, K_1.3375_ and K_actual_ in the untreated controls, post-LASIK/PRK and keratoconus group.

Pairing	Mean difference ± SD	95%CI	*P* Value
Untreated controls			
K_cal_- K_actual_	0.00 ± 0.11	-0.02 to 0.02	1.000
K_1.3315_- K_actual_	0.21 ± 0.11	0.19 to 0.22	<0.001
K_1.3375_- K_actual_	0.99 ± 0.12	0.97 to 1.00	<0.001
Post-LASIK/PRK group			
K_cal_- K_actual_	-0.01 ± 0.20	-0.06 to 0.05	1.000
K_1.3315_- K_actual_	0.85 ± 0.18	0.80 to 0.89	<0.001
K_1.3375_- K_actual_	1.56 ± 0.16	1.50 to 1.59	<0.001
Keratoconus group			
K_cal_- K_actual_	0.03 ± 0.67	-0.19 to 0.25	1.000
K_1.3315_- K_actual_	0.56 ± 0.70	0.33 to 0.79	<0.001
K_1.3375_- K_actual_	1.40 ± 0.76	1.15 to 1.64	<0.001

K_actual_ = actual total corneal power calculated based on Gaussian optics formula; K_cal_ = total corneal power calculated with the mean keratometric indices (1.3299 in untreated controls, 1.3242 in post-LASIK/PRK group and 1.3277 in keratoconus group); K_1.3315_ = total corneal power calculated with the keratometric index of 1.3315; K_1.3375_ = total corneal power calculated with the keratometric index of 1.3375.

**Fig 4:**
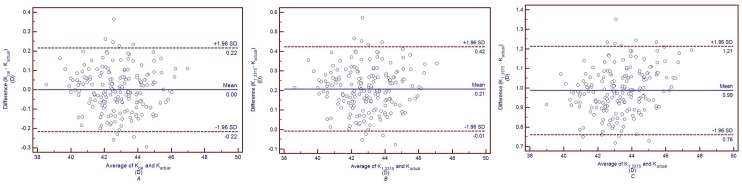
Band-Altman plots comparing the total corneal powers calculated with different indices (K_cal_, K_1.3315_ and K_1.3375_) and the actual total corneal power calculated based on Gaussian optics formula (K_actual_) in untreated controls. The solid lines represent mean differences, and the dotted lines represent 95%LoA. *A*: Comparison between K_cal_ and K_actual_. *B*: Comparison between K_1.3315_ and K_actual_. *C*: Comparison between K_1.3375_ and K_actual_. Kcal was calculated with the mean keratometric index of 1.3299 obtained in this study. K_1.3315_ was calculated with the keratometric index of 1.3315 obtained from the Gullstrand schematic eye. K_1.3375_ was calculated with the conventional keratometric index of 1.3375.

Fig 4 shows the Bland-Altman plots comparing the total corneal power calculated with different keratometric indices (K_cal_, K_1.3315_ and K_1.3375_) and the actual total corneal power calculated based on Gaussian optics formula (K_actual_) in untreated controls, and the 95% confidence interval (CI) for K_cal_ vs K_actual_, K_1.3315_ vs K_actual_, and K_1.3375_ vs K_actual_ were -0.22 to 0.22 D, -0.01 to 0.42 D and 0.76 to 1.21 D, respectively. In post-LASIK/PRK group, K_1.3315_ and K_1.3375_ were 0.85 ± 0.18 D and 1.56 ± 0.16 D lager than K_actual_, respectively (both *P*<0.05).

**Fig 5:**
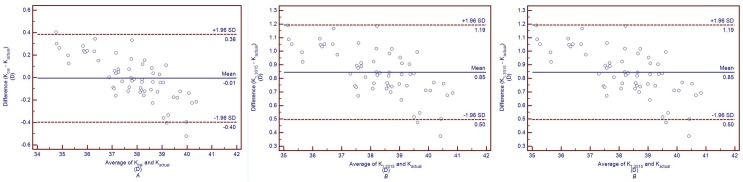
Band-Altman plots comparing the total corneal powers calculated with different indices (K_cal_, K_1.3315_ and K_1.3375_) and the actual total corneal power calculated based on Gaussian optics formula (K_actual_) in post-LASIK/PRK group. The solid lines represent mean differences, and the dotted lines represent 95%LoA. *A*: Comparison between K_cal_ and K_actual_. *B*: Comparison between K_1.3315_ and K_actual_. *C*: Comparison between K_1.3375_ and K_actual_. Kcal was calculated with the mean keratometric index of 1.3242 obtained in this study. K_1.3315_ was calculated with the keratometric index of 1.3315 obtained from the Gullstrand schematic eye. K_1.3375_ was calculated with the conventional keratometric index of 1.3375.

Fig 5 shows the Bland-Altman plots of K_cal_ and K_actual_, K_1.3315_ and K_actual_, K_1.3375_ and K_actual_ in post-LASIK/PRK group, and the 95% CI for K_cal_ vs K_actual_, K_1.3315_ vs K_actual_, and K_1.3375_ vs K_actual_ were -0.40 to 0.38 D, 0.50 to 1.19 D and 1.23 to 1.86 D, respectively. In keratoconus group, K_1.3315_ and K_1.3375_ were 0.56 ± 0.70 D and 1.40 ± 0.76 D lager than K_actual_, respectively (both *P*<0.05).

**Fig 6:**
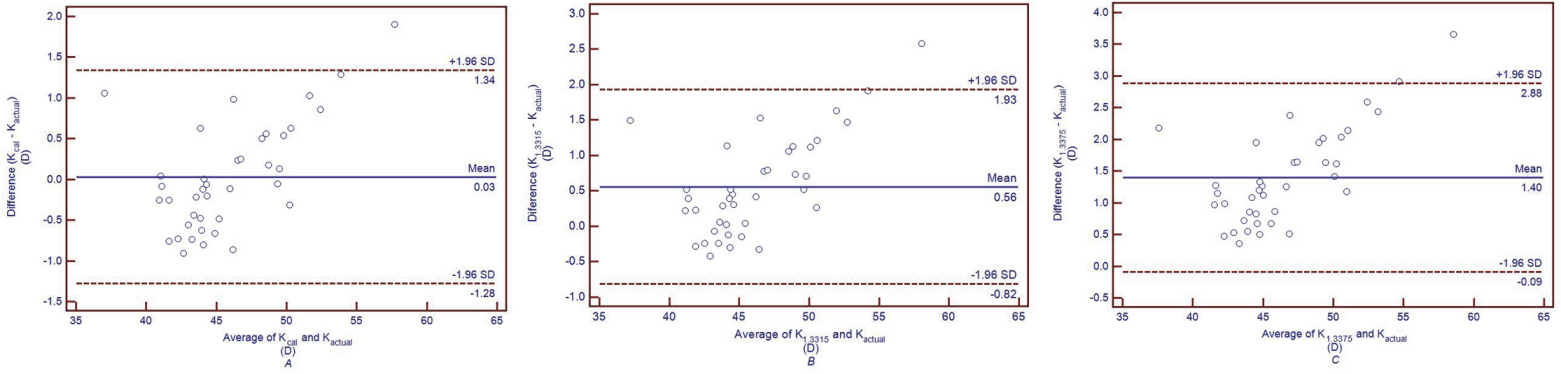
Band-Altman plots comparing the total corneal powers calculated with different indices (K_cal_, K_1.3315_ and K_1.3375_) and the actual total corneal power calculated based on Gaussian optics formula (K_actual_) in keratoconus group. The solid lines represent mean differences, and the dotted lines represent 95%LoA. *A*: Comparison between K_cal_ and K_actual_. *B*: Comparison between K_1.3315_ and K_actual_. *C*: Comparison between K_1.3375_ and K_actual_. K_cal_ was calculated with the mean keratometric index of 1.3277 obtained in this study. K_1.3315_ was calculated with the keratometric index of 1.3315 obtained from the Gullstrand schematic eye. K_1.3375_ was calculated with the conventional keratometric index of 1.3375.

Fig 6 shows the Bland-Altman plots of K_cal_ and K_actual_, K_1.3315_ and K_actual_, K_1.3375_ and K_actual_ in keratoconus group, and the 95% CI for K_cal_ vs K_actual_, K_1.3315_ vs K_actual_, and K_1.3375_ vs K_actual_ were -1.28 to 1.34 D, -0.82 to 1.93 D and -0.09 to 2.88 D, respectively.

## Discussion

Keratometric index is critical in both the calculation of total corneal power and the prediction of intraocular lens power. Clinically, the commonly used keratometric indices include the conventional 1.3375 and the 1.3315 based on the Gullstrand schematic eye. However, the keratometric indices calculated in real eyes using Scheimpfulg camera system[1] and Slit-scan system[6] were smaller than 1.3375 and 1.3315. To the best to our knowledge, this is the first study to calculate keratometric index based on data derived from RTVue FD-OCT.

In our study, we used the RTVue FD-OCT to measure the anterior and posterior corneal curvature within central 3 mm zone and central corneal thickness in untreated, post-LASIK/PRK and keratoconus eyes. Then we used the data obtained to calculate the mean ratio of the anterior corneal curvature to the posterior corneal curvature (Ratio) and the mean calculated keratometric index (N_cal_) in each group. In untreated controls, it was shown that the Ratio was 1.176 ± 0.022, and N_cal_ was 1.3299 ± 0.00085. Jin et al.[17] calculated a Ratio of 1.223±0.028 and a keratometric index of 1.3280 ± 0.0011 in 352 Chinese untreated eyes, and a Ratio of 1.205±0.027 and a keratometric index of 1.3287 ± 0.0010 in 205 German untreated eyes using data derived from Pentacam HR. Ho et al[5] used the data obtained by Pentacam to calculate a Ratio of 1.223±0.034 and a keratometric index of 1.3281 ± 0.0018 in 221 Chinese untreated eyes. Fam et al[6] applied OrbscanⅡto measure 2429 Malaysian untreated eyes,and obtained a Ratio of 1.22±0.03 and a keratometric index of 1.3273 ± 0.0013. It demonstrated that Ratio calculated by parameters derived from RTVue FD-OCT in untreated controls in our study was obviously smaller than those calculated by parameters obtained by Pentacam and OrbscanⅡ, and simultaneously, N_cal_ in untreated controls in our study was larger than those calculated by parameters obtained by Pentacam and OrbscanⅡ. The main source for the differences was the different R_posterior_ obtained. The R_posterior_ from untreated controls in our study was 6.569 ± 0.265 mm, which is larger than 6.30±0.24 mm from Chinese eyes and 6.42±0.25 mm from German eyes in Jin et al’s study, 6.34±0.28 mm in Ho et al’s study and 6.46±0.26 mm in Fam et al’ study. While the anterior corneal curvatures are comparable, a larger posterior corneal curvature will leads to a smaller ratio of anterior curvature to posterior curvature. Consequently, a larger total corneal power will be calculated based on a larger posterior corneal curvature (Formula 2), thus a larger N_cal_ will be calculated (Formula 3). The differences of R_posterior_ obtained by RTVue FD-OCT, Pentacam Scheimpflug camera system and Slit-scan system RTVue maybe caused by distinct measurement principles on which each device is based. RTVue FD-OCT with a speed of 26000 axial scans per second has an axial resolution of 5 μm. The corneal mapping model contained 6.0 mm line scans on 8 meridians with 1019 axial scans centered on the pupil, and the whole scan model was finished within 0.32 seconds. [16,20] The Pentacam using a rotating Scheimpflug camera to image the anterior segment, provides the anterior and posterior corneal surfaces, pachymetry maps. And it measures 50000 or 125000 data points in less than 2 seconds.[21–23] The OrbscanⅡis based on a slit-scan combining Placido ring technology. The data collected are processed to generate approximately 9000 data points over a 10 mm corneal diameter, and the Placido ring reflections are used to supplement slit-scan data to generate curvature-based map.[6] Furthermore, both ethnicity and average age of subjects in different studies maybe factors which contributed to the different R_posterior_ obtained.[24]

In our study, it was shown that Ratio from post-LASIK/PRK group (1.314 ± 0.042) was the largest; followed by Ratio from keratoconus group (1.229 ± 0.118); whereas Ratio from untreated controls was the smallest (1.176 ± 0.022) (all *P*<0.05). On the contrary, N_cal_ from post-LASIK/PRK group (1.3242 ± 0.00171) was the smallest; followed by N_cal_ from keratoconus group (1.3277 ± 0.0046); whereas N_cal_ from untreated controls was the largest (1.3299 ± 0.00085). We further analyzed the correlation and linear regression between Ratio and N_cal_ in each group. It demonstrated that N_cal_ was negatively correlated to Ratio in each group (N = 1.3752–0.03852 × Ratio (*r* = -0.997, *r* ^*2*^ = 0.995, P<0.05) (Fig 3*B*),N = 1.3775–0.04054 × Ratio (*r* = -0.999, *r* ^*2*^ = 0.999, P<0.05) (Fig 3*D*),N = 1.3759–0.03917 × Ratio (*r* = -0.999, *r* ^*2*^ = 0.999, P<0.05) (Fig 3*F*). Here, we can try to explain the reason why N_cal_ was closely negatively correlated to Ratio from the origin of the 2 parameters. We can combine formula 2 and formula 3 in Patients and Methods part. Then we obtained the equation as follows:

$$\begin{array}{l} \left({\text{n}}_{1}–{\text{n}}_{0}\right)/{\text{R}}_{\text{anterior}}+\left({\text{n}}_{2}–{\text{n}}_{1}\right)/{\text{R}}_{\text{posterior}}–\left(\text{CCT}/{\text{n}}_{1}\right)\times \left[\left({\text{n}}_{1}–{\text{n}}_{0}\right)/{\text{R}}_{\text{anterior}}\right]\times \\ \left[\left({\text{n}}_{2}–{\text{n}}_{1}\right)/{\text{R}}_{\text{posterior}}\right]=\left(\text{n}-1\right)/{\text{R}}_{\text{anterior}} \end{array}$$

where n_0_ (1.000), n_1_ (1.376) and n_2_ (1.336) are the refractive indices of the air, the cornea and aqueous, respectively.

The equation can be simplified as:

$$n=1.376+(0.011\times {\text{CCT)/R}}_{\text{posterior}}-0.04\times \text{Ratio}$$

In the simplified formula above, the value of (0.011×CCT)/R_posterior_ was very small. We assumed that CCT and R_posterior_ were 520 um and 6.5mm, respectively, then the value of (0.011×CCT)/R_posterior_ was calculated as 0.00088, which could be ignored comparing the constant of 1.376 in the same formula. Thus, we obtained a new formula: n = 1.376–0.04 × Ratio, which was very similar to the regression formulas in untreated, post-LASIK/PRK and keratoconus group. It means that N_cal_ and Ratio obtained from untreated, post-LASIK/PRK and keratoconus eyes have excellent similar linear relationship.

In our study, the total corneal powers were calculated using N_cal_ in each group (K_cal_), the keratometric index of 1.3315 derived from Gullstrand schematic eye (K_1.3315_) and the conventional keratometric index of 1.3375 (K_1.3375_). And we also compared K_cal_, K_1.3315_ and K_1.3375_ to the total corneal powers calculated based on Gaussian optics formula (K_actual_). In all the 3 groups, K_cal_ and K_actual_ were comparable (all *P* = 1.00). K_1.3315_ was 0.21 ± 0.11 D larger than K_actual_ in untreated controls, and the difference increased to 0.56 ± 0.70 D in keratoconus eyes, and the difference was 0.85 ± 0.18 D. Similarly, K_1.3375_ were 0.99 ± 0.12D, 1.40 ± 0.76D and 1.56 ± 0.16 D larger than K_actual_ in untreated, keratoconus and post-LASIK/PRK eyes, respectively. In Ho et al’s study[5] in which Pentacam Scheimpflug camera system was applied, K_cal_ and K_actual_ were also comparable in untreated controls, but the 95% CI was larger than that in our study (-0.46 D to 0.46 D in Ho et al’s, -0.21 D to 0.21 D in ours). The differences between K_1.3315_ and K_actual_, K_1.3375_ and K_actual_ were larger than those in our study (0.85 D and 1.21 D in Ho et al’s study). And also the 95% CI were larger than ours (-0.03 D to 0.89 D and 0.75 D to 1.67 D in Ho et al’s study; -0.01 to 0.42 D and 0.76 to 1.21 D in ours). It demonstrated that the agreement between K_cal_ and K_actual_ obtained by RTVue FD-OCT was better than that the one obtained by Pentacam Scheimpflug camera system in untreated eyes. In Wang et al’s study[25] using Galilei Scheimpflug camerasy system to obtain data from untreated eyes and myopic-LASIK/PRK eyes, K_1.3375_ was 1.30 D larger than K_actual_ in untreated eyes, and the difference increased to 1.51 D in eyes after corneal refractive surgery. In Jin et al’s study[17], K_1.3375_ was 1.26 D larger than K_actual_ in untreated eyes, and the difference increased to 1.71 D in eyes after corneal refractive surgery. Table 4 showed the differences between K_1.3375_ and K_actual_ obtained by distinct devices in untreated eyes and post-LASIK/PRK eyes. In our study, we found that the trends of the differences between K_1.3315_ and K_actual_, K_1.3375_ and K_actual_ coincided with the trend of Ratio, and opposited to the trend of calculated keratometric index in the 3 groups.

**Table 4:** Comparison of K_1.3375_ and K_actual_ in published studies.

Studies	Age	Eyes	Corneas	Device for K_1.3375_/K_actual_	K_1.3375_-K_actual_(D)
Borasio et al[1]	-	143	Untreated	Topcon /Petacam	1.30
Savini et al[12]	54.9±22	71	Untreated	Pentacam	1.25
Tang et al[15]	-	32	Untreated	Atlas /OCT	1.13
Wang et al[25]	36±11	94	Untreated	Galilei	1.30
	38±9	61	post-LASIK/PRK	Galilei	1.51
Jin et al[17]	46.05±21.05	352	Untreated	Pentacam HR	1.23
	-	102	post-LASIK/PRK	Pentacam HR	1.71
Hua et al[18,19]	-	77	Untreated	RTVue OCT	1.01
	24.5 ± 5.2	58	post-LASIK	RTVue OCT	1.55
Present study	26.1 ± 5.6	186	Untreated	RTVue OCT	0.99
	27.3 ± 6.2	60	post-LASIK/PRK	RTVue OCT	1.56

K_1.3375_: Keratometry calculated using the conventional keratometric index of 1.3375, K_actual_: Keratometry calculated based on Gaussian optics formula.

The limitations of this study included: 1) a small number of eyes were included in keratoconus group; 2) the repeatability of corneal parameters obtained by RTVue FD-OCT in keratoconus group has not been proved; 3) For post-LASIK/PRK group, only eyes after myopic-LASIK/PRK were included.

In conclusion, the ratio of anterior corneal curvature to posterior corneal curvature obtained by RTVue FD-OCT in untreated eyes is smaller than those obtained from Scheimpflug camera system and Slit-scan system in published studies, and also the calculated keratometric index obtained by RTVue FD-OCT in untreated eyes is larger than those obtained from Scheimpflug camera system and Slit-scan system in published studies. The calculated keratometric index was negatively related to the ratio of anterior corneal curvature to posterior corneal curvature in untreated, post-LASIK/PRK, and keratoconus eyes. Using the calculated keratometric index may improve the prediction accuracies of total corneal powers in untreated eyes, but not in post-LASIK/PRK and keratoconus eyes.
